# Measuring Sleep Health Disparities with Polysomnography: A Systematic Review of Preliminary Findings

**DOI:** 10.3390/clockssleep4010009

**Published:** 2022-02-18

**Authors:** Faustin Armel Etindele Sosso

**Affiliations:** Department on Global Health and Ecoepidemiology, Redavi Institute, Montréal, QC H4J 1C5, Canada; faustin.armel.etindele.sosso@umontreal.ca

**Keywords:** polysomnography, socioeconomic status, sleep, health disparities, systematic review

## Abstract

Socioeconomic status (SES) has an unrecognized influence on behavioral risk factors as well as public health strategies related to sleep health disparities. In addition to that, objectively measuring SES’ influence on sleep health is challenging. A systematic review of polysomnography (PSG) studies investigating the relation between SES and sleep health disparities is worthy of interest and holds potential for future studies and recommendations. A literature search in databases was conducted following Prisma guidelines. Search strategy identified seven studies fitting within the inclusion criteria. They were all cross-sectional studies with only adults. Except for one study conducted in India, all of these studies took place in western countries. Overall emerging trends are: (1) low SES with its indicators (income, education, occupation and employment) are negatively associated with PSG parameters and (2) environmental factors (outside noise, room temperature and health worries); sex/gender and BMI were the main moderators of the relation between socioeconomic indicators and the variation of sleep recording with PSG. Socioeconomic inequalities in sleep health can be measured objectively. It will be worthy to examine the SES of participants and patients before they undergo PSG investigation. PSG studies should always collect socioeconomic data to discover important connections between SES and PSG. It will be interesting to compare PSG data of people from different SES in longitudinal studies and analyze the intensity of variations through time.

## 1. Introduction

Sleep is an ensemble of recurrent biophysical processes that may be disturbed by a wide range of biological, psychological and external factors [1,2]. Among multiple stressors affecting sleep health, there is the individual’s socioeconomic status which is also associated to health disparities, as was previously reported for cardiovascular and metabolic diseases. Socioeconomic status (SES) is a latent concept of an individual’s economic and socioecological situation [3,4,5,6]. SES is a complex assessment of a socio-ideological and theoretical construct measured in a variety of ways usually taking into account several indicators such as employment, income, education, occupation and social position [3,4,5,6].

The majority of studies investigating sleep disturbances use self-reported instruments such as the Insomnia Severity Index (ISI) and the Pittsburgh Sleep Quality Index (PSQI), while very few studies in sleep research and health disparities have used objective measurement like actigraphy and polysomnography as an assessment tool [7,8]. Polysomnography involves the recording of several variables such as the electrical activity of the brain via electroencephalography, muscle activity via electromyogram and eyeball activity via electrooculogram [9]. Polysomnography also monitors sleep stages and cycles to identify if, why and when sleep patterns are disrupted [9,10]. In the context of sleep-wakefulness disorders and the majority of sleep disturbances, it is also a test of choice for both diagnostic and monitoring purposes [9,10] with parameters such as sleep efficiency and sleep continuity being measured.

An extensive screening of empirical literature revealed that no systematic review on the relation between socioeconomic status (SES), sleep health and its clinical measurement with polysomnography has been previously conducted. The goals of this systematic review are to (1) analyze how sleep health disparities are measured with polysomnography in the general population; and (2) suggest improvement for clinical practice.

## 2. Results

### 2.1. Characteristics of Studies

Seven studies [8,11,12,13,14,15,16] were identified and included in the final selection, all cross-sectional (Table 1). Three studies [9,10,12] were performed in the USA, two studies [8,16] performed in Switzerland, one study [13] in India and one study [15] in Brazil. The participants were all adults from the general population representing a global sample size of 7638 people. The smaller sample size was 128 [14] and the biggest was 3391 [8]. Participants’ age ranged from 18 years [14] to 81 years old [8,16]. The most used socioeconomic indicators were education in five studies [9,10,12,14,15], income (annual, household and financial strain) in three studies [9,10,13], occupation and occupational position in two studies [8,16], composite score/perceived SES in two studies [11,13] and employment in one study [15].

### 2.2. Polysomnography, Socioeconomic Indicators and Sleep Health

Sleep parameters measured with PSG in these studies are sleep duration [9,10,12], sleep latency [9,10,12,14], sleep efficiency [9,10,12,14], WASO [9,10,12], sleep architecture [9,10,12], stage shifts [8], sleep continuity [12] and total sleep time [8]. The duration of PSG recording ranged from one [8,13,15,16] to three nights [12]. Findings showed that lower SES was associated with longer sleep latency [11], more WASO [11], lower sleep efficiency and higher stage shifts in PSG [8]. Individuals with lower childhood SES spent more time in Stage 2 sleep and less time in SWS than participants from higher childhood SES backgrounds independently of current SES [14]. Financial strain was a significant correlate of poorer subjective sleep quality and PSG-assessed sleep continuity [12]. Men with a low educational level or occupational position were more likely to suffer from poor sleep quality, short sleep duration and insomnia [8]. In addition, men with a low occupational position were also more likely to have long sleep latency [8]. Women with a low educational level were more likely to have long sleep latency and short sleep duration [8]. Women with a low occupational position were also more likely to have longer sleep latency and short sleep duration in addition of excessive daytime sleepiness [8].

### 2.3. Interactions and Moderators of Polysomnography Recording

Environmental factors (outside noise, room temperature and health worries) and negative effects were statistical mediators of the relationship between SES and PSQI scores [11]. Women from low childhood SES backgrounds had longer sleep latency than women from the high childhood SES group [14]. One study found that SES affects OSA risk differentially for males and females, with low income acting as a moderator in the relationship between SES and individual’s sleep [15]. Finally, one study reported that the association between home PSG measures of OSA and SES was mediated by BMI [16].

## 3. Discussion

### 3.1. Summary of Findings

Preliminary findings showed that all seven studies were all cross sectional, conducted on adults and performed in western countries except for one Indian study. Overall trends emerging are: (1) low SES with its indicators (income, education, occupation and employment) are negatively associated with PSG parameters and (2) environmental factors (outside noise, room temperature and health worries), sex/gender and BMI were the main moderators of the relation between socioeconomic indicators and the variation of sleep recording with PSG. OSA seems more of a mediating factor for sleep quality because OSA is more prevalent in low SES populations.

### 3.2. Relation with Current Knowledge

Over time, SES has been established as an important determinant of health but also as a mechanism for social inequalities between groups of individuals [17,18,19,20]. Recently, studies have revealed profound implications of SES for various social inequalities related to sleep disturbances [21,22,23]. However, we know very little about the role played by SES in the development and severity of sleep health disparities regardless of how these disparities are assessed. This review provided additional information on the impact of socioeconomic indicators on objective sleep measurement. PSG parameters are associated to a variation of global SES as well as variations of individual socioeconomic indicators. The direction of this association is similar to what has been previously reported with subjective assessment of sleep health disparities: People with low SES reported more sleep disturbances regardless of their age which is known as an important sleep modifier. Another important remark is the fact that all studies included in this review were performed in developed countries: three studies were performed in the USA, two studies were performed in Switzerland, one study in India and one study in Brazil. More studies performed in developing countries with members of the general population are necessary to draw the big picture of the currently studied relationship.

### 3.3. Improvements for Clinical Practice

There are many studies on SES sleep that have used self-reported questionnaires [24,25,26,27,28] and objective/validated tests [21,29,30,31,32] without any emerging consensus on this relation, adding to the great heterogeneity of SES measures for which there are no clear recommendations [33,34]. This contributes to the methodological difficulty of quantifying the association between SES and sleep health, which explains the lack of meta-analysis and the scarcity of systematic reviews on the subject for adults as well as the pediatric population (children and adolescents). The present study provides evidence that PSG can help identify individual differences in sleep health among people with or without a sleep disorder diagnosis confirmed by a physician or a sleep specialist. The following suggestions may improve future investigations:

PSG studies should always collect socioeconomic data to discover important connections between SES and PSG. The concept of “sleep health” is new in sleep research and public health. It includes several domains of sleep components such as sleep quality, sleep schedule and sleep disturbances. Sleep health is the global approach of sleep with its determinants, risk factors and implications for public health and different levels of policies. It would be important to analyze the SES of participants and patients before they undergo PSG investigation. Sleep can be altered by SES indicators and it would be interesting to compare PSG data of people from different SES in longitudinal studies and analyze the intensity of variations through time.

Promote use of PSG and actigraphy in SES research. It is easier and more conventional to use validated questionnaires or self-reported items to investigate health disparities; however, PSG as well as actigraphy provide very useful and accurate details, including sleep continuity or WASO, that can objectively indicate sleep disorders or sleep health disparities with more accuracy than subjective assessments [7]. More basic training and advanced lectures related to PSG and actigraphy should be provided to students as well as researchers with interest in sleep, regardless of their background or their expertise. A joint effort of academic communities and private industries can help provide PSG accessibility with affordable prices. A tool too expensive is not used by targeted individuals and is not profitable for the manufacturer.

## 4. Materials and Methods

### 4.1. Literature Search

**Figure 1:**
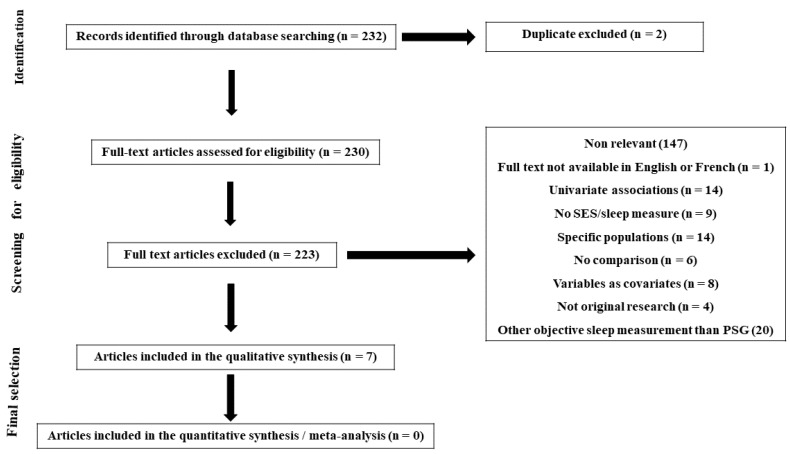
Prisma flowchart of study selection process: the relationship between SES and sleep health disparities measured by PSG.

Relevant citations for this review were identified by searching the databases PubMed/Medline and Google scholar between January 2000 and April 2021. A combination of search terms was used: “socioeconomic”, “socioeconomic status”, “socio-economic”, “social position”, “social class”, “socioeconomic position”, “sleep”, “sleep disorders”, “sleep disturbances”, “sleep complains”, “polysomnography”, “wake after sleep onset”, “time in bed *”, “sleep efficiency”, “sleep duration”, “sleep quality”, “sleep diary *” and “sleep fragmentation *”. All included articles were identified on the basis of relevance to the association between SES and polysomnography parameters following the PRISMA guidelines (Figure 1).

### 4.2. Inclusion and Exclusion Criteria

Observational studies were defined as of any design (cross-sectional, retrospective or longitudinal) that evaluated humans of any age, gender or race/ethnicity from the general population. The article had to include an objective measure of SES, such as education, income, assets, occupation, employment status and composite index, as well as perceived SES, self-reported by participants. Proxy measures of SES (neighborhood SES or area deprivation indices) were also included when individual data was not available. For studies examining children or adolescents, perceived family SES measures such as parental education, parental profession or household income were used instead. The investigation also needed to include a polysomnography-based measure of sleep and potential moderators such as AHI (apnea–hypopnea index). Studies were excluded based on the following criteria: (1) They were interventional trials, reviews or meta-analyses, case series or case reports and/or did not present original research, (2) they were not written in English or French, (3) the full text was not accessible, (4) authors/researchers recruited participants that already presented specific conditions at baseline (for example individual with cancer, shift-workers, children with cerebral palsy, etc.), (5) they did not provide statistical significance in cases where either SES or sleep were evaluated as covariates or mediators and (6) researchers used actigraphy instead of polysomnography.

## Figures and Tables

**Table 1:** Characteristics of included studies investigating the association between socioeconomic disparities in sleep health and polysomnography.

Study	Study Design	Population	Age (Mean ± SD or Range)	Sample Size	Socioeconomic Indicators	Sleep Health Measurement	Interactions and Moderators	Conclusions
[11]	Cross-sectional	Adult members of a larger cohort in Pittsburgh metropolitan area	45–75	187	Composite SES score (education and annual income)	Two-night home PSG (sleep duration, sleep latency, sleep efficiency, WASO, sleep architecture, apnea–hypopnea index (AHI))	Environmental factors (outside noise, room temperature and health worries) and negative effects were statistical mediators of the relationship between SES and PSQI scores	Lower SES was associated with longer sleep latency and more WASO
[12]	Cross-sectional	Midlife women from the general population of 4 US cities	50.72 ± 2.02	368	Educational attainment (college or advanced degree vs without). Financial strain (somewhat to very difficult paying for basics vs not difficult at all)	Three-night home PSG assessing sleep duration, sleep continuity, sleep latency, WASO, sleep efficiency, sleep architecture and power spectral analysis of NREM EEG	N/A	Financial strain was a significant correlate of poorer subjective sleep quality and PSG-assessed sleep continuity
[13]	Cross-sectional	Adults from the general population in South Delhi, India	30–65	360	Kuppuswami socioeconomic status score	OSA (AHI ≥ 5 in PSG)	N/A	Prevalence of OSA was not significantly different across the socio-economic strata
[14]	Cross-sectional	Adults recruited through advertisements in San Diego, California	18–52	128	Childhood SES: highest level of education attained by each parent (low if neither parent achieved education beyond high school, and high if either parent achieved some education beyond high school)	PSG (sleep duration, latency, efficiency, architecture, WASO)	Women from low childhood SES backgrounds had longer sleep latency than women from the high childhood SES background group	Individuals with lower childhood SES spent more time in Stage 2 sleep and less time in SWS than participants from higher childhood SES backgrounds independently of current SES
[15]	Cross-sectional	Adults from the general population in Sao Paulo, Brazil	20–80	1042	Annual household income (high, middle or low) according to the Brazilian Economic Classification Criteria Employment Status (working vs not working)	OSA ICSD-2 criteria (AHI from PSG)	Income affects OSA risk differentially for males and females	Global SES was not associated with OSA
[8]	Cross-sectional	Adults from the general population in Lausanne, Switzerland	40–81	3391	Educational level (high, middle, low). Occupational position (high, middle, low)	Total sleep time, sleep latency, slow wave sleep, sleep efficiency, stage shifts (in-home 1-night PSG)	N/A	Men with a low educational level or occupational position were more likely to suffer from poor sleep quality, short sleep duration and insomnia. Men with a low occupational position were also more likely to have long sleep latency. Women with a low educational level were more likely to have long sleep latency and short sleep duration. Women with a low occupational position were more likely to have long sleep latency, excessive daytime sleepiness and short sleep duration. Participants with low SES had lower sleep efficiency and higher stage shifts in PSG.
[16]	Cross-sectional	Adults of a general population cohort in Lausanne, Switzerland	40–81	2162	Occupation (managers, lower-level executives, low qualified non-manuals and manuals). Education (university, higher secondary, lower secondary or lower)	Home PSG (apnea–hypopnea index (AHI) and ≥ 3% oxygen desaturation index (ODI))	These associations were mediated by BMI	Lower occupational position was associated with an increased risk of AHI ≥ 30 and ODI ≥ 30. Lower education was associated with an increased risk of ODI ≥15.

SES, socio-economic status; PSQI, Pittsburgh Sleep Quality Index; BMI, body mass index; WASO, wake after sleep onset; OSA, obstructive sleep apnea; PSG, polysomnography; AHI, apnea–hypopnea index; NREM, non-rapid eye movement; EEG, electroencephalogram; SWS, slow-wave sleep; REM, rapid eye movement; RBD, REM sleep behavior disorder; ISI, Insomnia Severity Index; ODI, oxygen desaturation index.
